# High expression of ENPP1 in high-grade serous ovarian carcinoma predicts poor prognosis and as a molecular therapy target

**DOI:** 10.1371/journal.pone.0245733

**Published:** 2021-02-26

**Authors:** Hanzhi Wang, Feng Ye, Caiyun Zhou, Qi Cheng, Huaizeng Chen

**Affiliations:** 1 Women’s Reproductive Health Key Laboratory of Zhejiang Province, Women’s Hospital, School of Medicine, Zhejiang University, Hangzhou, Zhejiang, P. R. China; 2 Department of Pathology, Women’s Hospital, School of Medicine, Zhejiang University, Hangzhou, Zhejiang, P. R. China; Duke University School of Medicine, UNITED STATES

## Abstract

Recent studies have shown that the expression of ENPP1 is related to differentiation, death, dissemination and chemosensitivity of tumor cells. So far, there is no research in ovarian carcinoma. This study aimed at exploring the role of ENPP1 gene in ovarian carcinoma, the relationship with prognostic indicators and chemotherapy resistance, and investigates the possibility of molecular targeted therapy. The expression of ENPP1 in 41 normal ovarian epithelial tissues, 97 ovarian serous cystadenoma and 103 HGSOC tissues was detected by IHC. In ovarian cancer tissues and ovarian cancer cell lines, mRNA and protein expression of ENPP1 was determined by qRT-PCR and Western blot. The ENPP1 expression was knockdowned by siRNA. Cell proliferation was measured with the BrdU Cell Proliferation ELISA. Cell migration and invasion were detected by Wound-Healing, Transwell migration and Matrigel invasion assay. Caspase 3 activity was determined by the CaspACE. The expression of EMT markers such as E-cadherin, N-cadherin, and Vimentin was measured, and the expression of PCNA and MMP9 was also be detected. The results showed that the expression of ENPP1 was significantly increased in high-grade ovarian serous carcinoma, the number of strong expression was 85.4% (22.3%+63.1%) and only 1.03% (1.03%+0.0%) in serous cystadenoma, but no in normal ovarian epithelium (P< 0.05). And the stronger the expression of ENPP1, the later the FIGO stage and the poorer differentiation of cells (P = 0.001 or <0.001, respectively). However, no correlation was found between the expression of ENPP1 and chemosensitivity. ENPP1 was also highly expressed in ovarian cancer tissues and in epithelial ovarian cancer cell lines (A2780, CaoV3, OVCAR3, SKOV3 and 3ao). After down-regulation of ENPP1 expression by RNA interference, the cell proliferation, migration and invasion of ovarian cancer cell decreased significantly, the expression of apoptosis related gene caspase 3 increased significantly, while the expression of PCNA and MMP9 was significantly down regulated. In addition, EMT biological characteristics of A2780 and SKOV3 cells were also inhibited. In summary, the increased expression of ENPP1 may be related to the occurrence of HGSOC, and indicate that the disease progresses rapidly and the prognosis is poor. ENPP1 may be considered as a potential molecular therapeutic target.

## Introduction

Almost 240,000 women worldwide are diagnosed with ovarian cancer every year. Although the incidence of ovarian cancer ranks second in female malignant tumors, the total mortality rate is almost three times that of breast cancer, making it the most lethal gynecological malignancy in developed countries. Because there are no early clinical symptoms and signs, and there is no effective method for early screening and diagnosis, about 70% of patients have been diagnosed in advanced stage, and the prognosis is generally poor. The 5-year survival rate of patients with stage III or IV was less than 25% [1, 2]. In China, the incidence of ovarian cancer is increasing year by year due to the aging of society [3]. The mortality rate has also ranked to first in female tumors, and the incidence rate has a younger trend in recent years [4–6].

Both histological and molecular studies show that ovarian cancer is not only clinically diverse, but also highly heterogeneous in its histopathological and molecular biological mechanisms [7]. But the vast majorities (about 90%) of ovarian cancers are EOCs. Epithelial ovarian cancer can be divided into type I and type II. Type I tumors tend to grow slowly and often have identifiable precancerous lesions, while type II tumors are high-level, rapidly progressing diseases, and often do not have precancerous lesions [8, 9]. Of type II tumors, 75% are HGSOC, but unfortunately, it is also the most aggressive of all ovarian cancers and the fastest-growing [10, 11]. At present, there are no effective and specific indicators and methods for early prediction of HGSOC. At present, the main clinical screening methods include vaginal ultrasound, serum CA-125 detection, etc., but there are great limitations in clinical practice [12]. Even if the tumor cells have been widely spread and metastasized in the abdominal cavity, they lack specific symptoms and cannot be detected and diagnosed early. Similarly, there are no specific and sensitive reference indicators for chemosensitivity and prognosis.

Ovarian cancer is a complex disease caused by multiple factors and multiple genes. Each pathological type has different molecular origins, and even within the same type, there are different molecular origins [7]. This leads to a great difference in molecular and cellular behavior, clinical manifestations and prognosis. At present, clinical treatment and prognosis can only be judged according to pathological stage, which is totally divorced from the basis of molecular biology and cannot provide an effective basis for stratified treatment based on molecular and cytological levels, leading to unsatisfactory diagnosis and treatment outcome [11]. In practice, more precise markers are urgently needed to classify patients into high-risk and low-risk types, so that effective personalized treatment can be adopted to improve the treatment effect and prognosis.

ENPP1 is a type II transmembrane glycoprotein with pyrophosphatase and phosphodiesterase activities. It is highly expressed in bone and cartilage. ENPP1 hydrolyzes pyrophosphate (as in ATP) and phosphodiester (as in oligonucleotides) to produce nucleoside 5 ’- monophosphate as part of nucleotide pyrophosphatase/phosphodiesterase (NPP) mediated function, which is essential in a variety of cellular processes including nucleotide cycling, purinergic receptor signal transduction and ATP mediated apoptosis [13]. The expression of ENPP1 in endometriosis, lung cancer cells, breast cancer cells and glioblastoma was significantly increased [14, 15]. So far, however, no research has been done in HGSOC.

In this study, we investigated the protein expression of ENPP1 in HGSOC. The results showed that the mRNA and protein expression of ENPP1 was significantly increased in HGSOC and was significantly correlated with poor prognosis. ENPP1 protein not only played an important role in the pathogenesis of HGSOC, but also played an important role in the malignant progression of HGSOC. After down-regulating the expression of ENPP1, the proliferation, migration and invasion of ovarian cancer cells were significantly inhibited, suggesting that ENPP1 could be a potential molecular therapeutic target.

## Materials and methods

### Study patients

A total of 241 specimens of normal ovarian tissue, ovarian serous cystadenoma and HGSOC from January 2004 to December 2010 were collected from Women’s Hospital, School of Medicine, Zhejiang University. The samples included 41 normal ovarian epithelial tissues, 97 ovarian serous cystadenoma and 103 HGSOC (including 6 cases of I stage, 30 cases of II stage, 62 cases of III stage and 5 cases of IV stage). Preoperative chemotherapy, immunotherapy or radiotherapy were excluded. All patients underwent initial surgery followed by paclitaxel-based chemotherapy.

The following clinical indicators were collected for all patients: preoperative serum CA125, ascites volume, maximum diameter of tumors, FIGO stage, cell differentiation grade and chemosensitivity. Patients with progressive disease during chemotherapy or relapse within 6 months after the completion of the initial chemotherapy course were identified as chemoresistantance. Patients with recurrence over 6 months or without recurrence were defined as chemosensitivity.

Freshly frozen tumor tissues of 21 normal ovarian epithelial tissues, 32 ovarian serous cystadenomas and 36 HGSOC were used to detect protein expression by Western Blot or mRNA expression by qRT-PCR.

The study was approved by the Ethics Committee of the Faculty of the Affiliated Women’s Hospital, School of Medicine, Zhejiang University (approval number: 2004002). All patients signed the informed consent. And all method protocols were carried out in accordance with the approved guidelines and regulations.

### Detection of ENPP1 protein expression by IHC

The ENPP1 protein expression in high grade serous ovarian carcinoma, ovarian serous cystadenoma and normal ovarian epithelium was detected by IHC. Fixed with 10% formalin, dehydrated with alcohol, and embedded in paraffin, the tissue was sliced into 4 micron thick sections on the slicer. The slides were dewaxed by methanol and rehydrated. 0.01M citrate buffer (pH = 6.0) was heated to boiling by microwave, the above-mentioned hydrated tissue sections were put into boiling buffer, the antigens were retrieved by microwave treatment for 10min. Each slide was incubated at room temperature for 10min with 1 drop of 3% hydrogen peroxide to block the activity of endogenous peroxidase. Add goat serum sealant and react at room temperature in wet box for 10min. Mouse monoclonal antibody (1:500) was added to cover all tissues and placed in a wet box at room temperature for 60min. Secondary antibody labeling, colouring and re-staining were performed using DAKO’s EnVision (+) System kit (Cat.K5007, Dako Diagnostica, Hamburg, Germany). The slides were counterstained by hematoxylin. Negative controls were obtained by replacing the primary antibody with normal bovine serum. At last, the expression intensity of ENPP1 and the number of positive cells were observed and recorded under the microscope.

### Protein expression intensity and scoring criteria

The presence of brown-yellow granules in the cytoplasm and membrane of tumors is considered to be positive. The expression of ENPP1 protein was semi-quantitatively evaluated according to the percentage of positive cells and staining intensity (400×, 10 random fields). Negative staining was defined as 0 points, light yellow as weak staining 1 point, brown yellow as medium staining 2 points, dark yellow as strong staining 3 points. When the percentage of positive cells was scored, <5% was defined as 0 point, <25% was defined as 1 point, <75% was defined as 2 points, and>75% was defined as 3 points. The total score was obtained by multiplying the intensity and percentage score. Each section was independently scored by two pathologists. The results of semi-quantitative analysis were as follows: negative—(0 points), weak positive + (1–2 points), moderate positive ++ (3–4 points) and strong positive +++ (5–6 points) [16].

### Cell lines and culture conditions

Ovarian epithelial cancer cell line A2780, CaoV3, OVCAR3, SKOV3 and 3ao are preserved by Women’s Reproductive Health Key Laboratory of Zhejiang Province. All of cell lines were maintained in RPMI 1640 culture medium, supplemented with 10% fetal bovine serum (FBS) at 37°C and 5%CO_2_ incubator.

### Western blot analysis

Ovarian epithelial cancer cell lines or frozen tumor tissues of 21normal ovarian epithelial tissues and 36 HGSOC were selected to detect the protein expression. Total protein was extracted from cell lines or tissues using RIPA lysis buffer. 10ul (5ug/ul) of each sample was loaded into an 8% PAGE. Subsequently, proteins were transferred to a 0.45 um PVDF membrane. After blocking in 5% non-fat milk for 1h, membranes were incubated with primary antibodies: mouse monoclonal ENPP1 antibody (Cat.sc-166649, Santa Cruz Biotech, 1:2000) or mouse monoclonal GAPDH antibody (Cat.60004-1-Ig, Proteintech, 1:5000) for 4°C overnight. Membranes were then washed with TBS containing 0.05%Tween-20 followed by a 2h incubation with an goat anti-mouse HRP-conjugated secondary antibody (Cat.SA00001-1, Proteintech, 1:5000). After a final wash, the membranes were imaged using an Image Quant LAS 4000 mini (GE Healthcare) with ECL (Cat.sc-2048, Santa Cruz Biotech). Then quantitative analysis of the proteins was performed.

### mRNA expression by qRT-PCR

Cell lines or freshly frozen tumor tissues of 21normal ovarian epithelial tissues, 32 ovarian serous cystadenoma and 36 HGSOC were selected. Total RNA was extracted using Trizol reagent (Cat.15596-026, Invitrogen, USA) according to the manufacturer’s protocol. Total RNA was treated with RNase-free DNase I. cDNA was reversed transcription and used as a template for qPCR detection. The following PCR primer pairs were used for quantitative amplification; 95°C 30s, 40 cycles at 95°C 5s followed with 60°C 30s. The primers of ENPP1 (mRNA: NM_006208.3) were 5’- GTCGTCAGTGGTCCTGTGTT -3’; 5’- TGCAAAGGCGTCTGAGATGT-3’. The primers of PCNA (mRNA: NM_002592.2) were 5’- CAGAGCTCTTCCCTTACGCA -3’; 5’- GTCCTTGAGTGCCTCCAACA-3’. The primers of MMP9 (mRNA: NM_004994.3) were 5’- CGACGTCTTCCAGTACCGAG -3’; 5’- GTTGGTCCCAGTGGGGATTT-3’. GAPDH was used as internal control. The primers of E-cadherin (mRNA: NM_001317184.2) were 5’- GCTGGACCGAGAGAGTTTCC -3’; 5’- CAAAATCCAAGCCCGTGGTG -3’. The primers of N-cadherin (mRNA: NM_001308176.2) were 5’- ATCCCTGCTTTCATTCTGACA -3’; 5’- CAGTTGCTAAACTTCACTGAAAGG -3’. The primers of Vimentin (mRNA: NM_003380.5) were 5’- GCAGGAGGCAGAAGAATGGT -3’; 5’- CGTTCCAGGGACTCATTGGTT-3’. The primers of GAPDH (mRNA: NM_001256799.2) were 5’-GAGAAGGCTGGGGCTCATTT-3’; 5’-AGTGATGGCATGGACTGTGG-3’. The PCR product length of ENPP1, PCNA, MMP9, E-cadherin, N-cadherin, Vimentin and GAPDH were 164bp, 200bp, 189bp, 155bp, 176bp, 174bp and 231bp, respectively. All reactions were performed with a ViiA 7 Dx System (ABI). The cutoff point (Ct) was defined as the value when the fluorescent signal increased above the background threshold. The ΔCt for gene-specific mRNA expression was calculated relative to the Ct of GAPDH. Relative mRNA expression was calculated with the formula: 2^-ΔCt^.

### Knockdown of the ENPP1 expression by RNA interference

In all the experiments, including cell proliferation, wound healing, migration, Matrigel invasion assay, and caspase 3 activity, ENPP1 expression was transiently downregulated by ENPP1 siRNA. Ovarian epithelial cancer cell lines were transfected with PC-1 siRNA (Cat.sc-40811, Santa Cruz Biotech) (PC-1 is namely ENPP1 gene, and the PC-1siRNA mentioned later represents ENPP1 siRNA) or Control siRNA (Cat.sc-37007, Santa Cruz Biotech) as scramble control for 48h using the Lipofectamine 3000 transfection reagent (Cat.L3000008, Invitrogen, USA) according to the protocol provided by the manufacturer.

### Cell proliferation inhibition assay

Cell proliferation was measured with the BrdU Cell Proliferation ELISA Kit (colorimetric) (Cat. 11647229001, Roche). Absorbance (A) was measured at 370 nm wavelength (reference 492 nm), and the calculation formula was as follows: A_siRNA_/A_Blank_.

### Wound-healing migration assay

A2780 cells and transfected A2780 cells were seeded onto six-well plates. When cells grew close to 85% confluence, a scratch was made using sterile 200 μL plastic pipette tips, and then cells were washed twice with PBS. After washing, cells were cultured in complete media. Images of wound closure were taken photo by an inverted microscope at 0h, 24h and 48h post wounding. All experiments were in triplicate.

### Transwell migration/matrigel invasion assay

After transfection for 48h, A2780 cell migration and invasion were determined using Transwell Permeable Supports with a pore size of 8 μm (Cat.3428, Corning) without or with Matrigel (Cat.356234, BD Biosciences). After serum starvation for 24h, the transfected cells that were resuspended (5×10^4^ cells per well) in 200μL of serum-free media were plated in the upper chambers. The lower transwell chamber contained the chemoattractant (10% FBS). For the invasion assay, the upper transwell chamber was coated with Matrigel, and for the migration assay, the chamber was uncoated. After incubated at 37°C and 5% CO_2_ for 24h, the upper surface of the membranes was then gently scraped using cotton swabs and washed with PBS to remove the stationary cells. Then, the membranes were fixed in 95% ethanol for 25min followed by staining with hematoxylin. The number of migrated cells was counted and averaged between ten random fields per well.

### Caspase 3 activity detection

After cell transfection for 24h, cells were collected and adjusted to 1×10^8^ cells/ml. Cells were lysed for 15min, and the lysate was centrifuged for 20min at 15,000×g to collect the supernatant. The Caspase 3 activity was determined at 405 nm by the CaspACE Assay System (colorimetric) kit (Cat.G7351, Promega) according to manufacturer’s instructions.

### Statistical analysis

The expression of ENPP1 was evaluated by Kruskal-Wallis H and Mann-Whitney U tests. Spearman and Kendall tests were used to analyze the relationship between the positive expression of HGSOC and clinicopathological factors. The data of all results are presented with mean ± standard deviation, and the statistical significance was determined by ANOVA with a post hoc analysis (Fisher least significant difference) or a Student’s two-tailed t-test (as the case may be). When the P value was <0.05 bilaterally, the results were considered to be statistically significant. All the statistical work was performed in SPSS software ver18.0 for Windows OS.

## Results

### The expression of ENPP1 and its correlation with normal ovarian tissue, ovarian serous cystadenoma and HGSOC

Based on the experimental results, we found that ENPP1 protein was mainly expressed in the cell membrane and cytoplasm. ENPP1 protein was negative in normal ovarian epithelial tissues, negative or weak positive in ovarian serous cystadenoma, and highly and strongly positive in HGSOC. The number of cases with strong expression was 85.4% (22.3% +63.1%) and only 1.03% (1.03% +0.0%) in serous cystadenoma, but not in normal ovarian epithelium. There were significant differences between the two groups each other. This result can be used to differentiate ovarian serous carcinoma from serous cystadenoma.

qRT-PCR results also showed that the expression of ENPP1 mRNA in HGSOC was significantly higher than that in normal ovarian tissue and ovarian serous cystadenoma (t = 18.423, P = 0.000)

**Fig 1:**
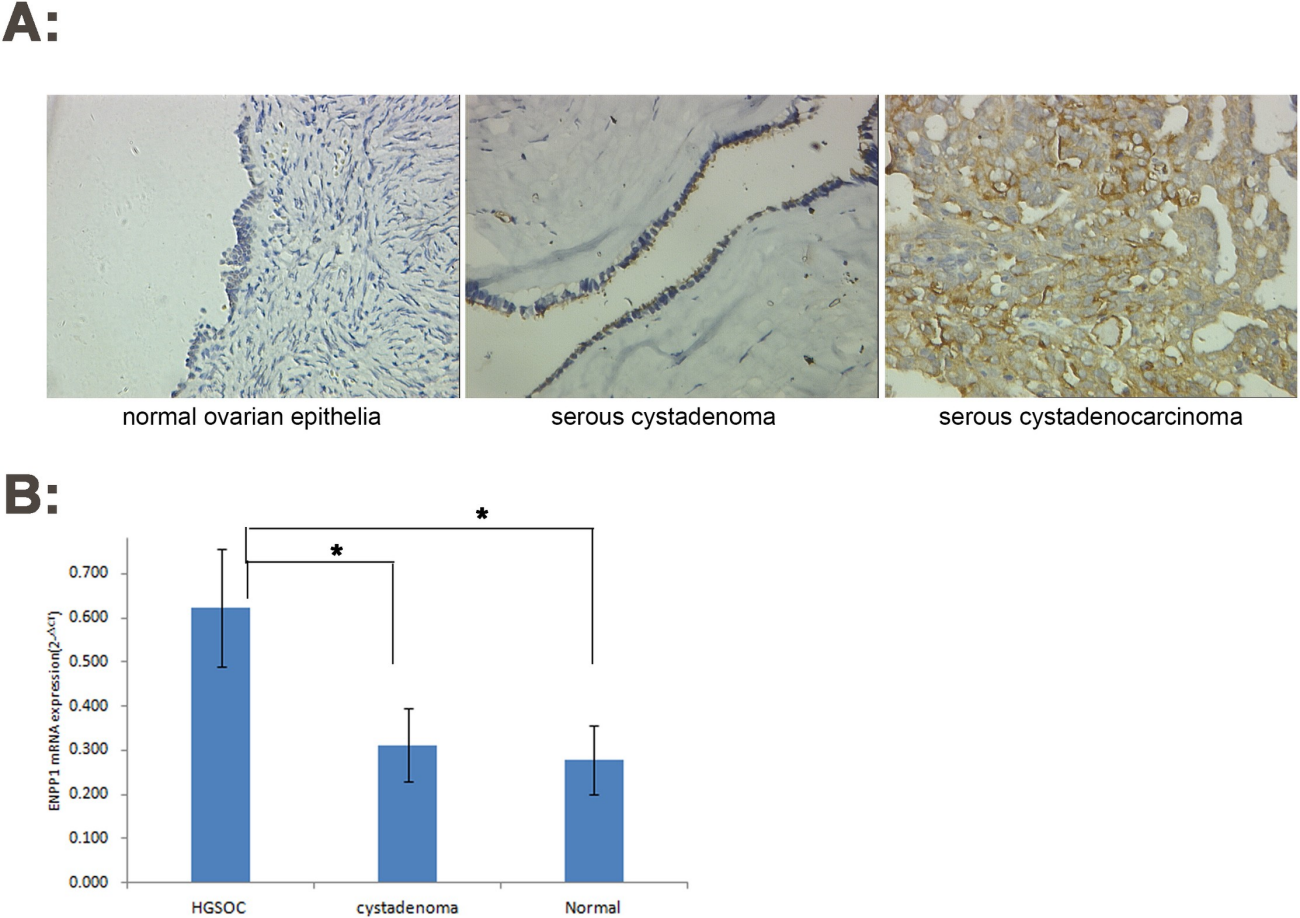
Increased expression of ENPP1 in high-grade serous ovarian carcinoma. A: IHC (×200); B: qRT-PCR. *P<0.05.

Data and statistical results are shown in detail in Fig 1 and Table 1.

**Table 1:** Expression of ENPP1 protein in normal ovarian tissue, ovarian serous cystadenoma and high-grade serous ovarian carcinoma.

	Cases	Protein expression intensity				Z/χ^2^	P
	241	(-)	(+)	(++)	(+++)		
normal ovarian epithelia	41	40	1	0 (0.0%)	0 (0.0%)	188.037	0.000^#^
serous cystadenoma	97	79	17	1 (1.03%)	0 (0.0%)	-0.2505	0.012*
serous cystadenocarcinoma	103	3	12	23 (22.3%)	65 (63.1%)	-9.657	0.000**
						-12.158	0.000***

# Comparison among three groups

*normal ovarian epithelia VS serous cystadenoma

** normal ovarian epithelia VS serous cystadenocarcinoma

***serous cystadenoma VS serous cystadenocarcinoma.

### Stratified analysis of clinicopathological prognostic factors and chemosensitivity of ENPP1 protein expression in HGSOC

We stratified the relationship between prognostic parameters, chemosensitivity and ENPP1 expression in patients with high-grade serous ovarian carcinoma. We found that 98.4% (24.2% +74.2%) and 100% (60.0% +40.0%) were strongly expressed in patients with stage III and IV of FIGO stage, respectively, while 73.4% (16.7% +56.7%) in patients with stage II of FIGO stage. There were no cases of strong expression in patients with stage I. At the same time, we found that with the decrease of tumor cell differentiation, the number of cases with strong ENPP1 expression gradually increased. In patients with cell differentiation grade I, grade II and grade III, the proportion of cases with strong expression was 12.5% (12.5% +0.0%), 78.9% (44.7% +34.2%) and 100% (8.8% +91.2%) respectively. These results showed that the stronger the expression of ENPP1, the later the FIGO stage and the poorer the differentiation of tumor cells. However, no correlation was found between the expression of ENPP1 and chemosensitivity in our analysis. Data and statistical results are shown in detail in **Table 2**.

**Table 2:** Relationship between ENPP1 protein expression, prognostic parameters and chemosensitivity in high-grade serous ovarian carcinoma.

		Cases	Protein expression intensity				r	P
		103	(-)	(+)	(++)	(+++)		
Preoperative serum CA125 (U/ml)								
	<500	43	1	5	9	28	-0.033	0.729
	≥500	60	2	7	14	37		
Volume of ascites (ml)								
	<500	63	0	7	13	43	-0.145	0.125
	≥500	40	3	5	10	22		
Tumor maximum size (cm)								
	≤10	56	2	6	12	36	-0.021	0.823
	>10	47	1	6	11	29		
FIGO stage								
	I	6	3	3	0 (0.0%)	0 (0.0%)	**0.310**	**0.001**
	II	30	0	8	5 (16.7)	17 (56.7)		
	III	62	0	1	15 (24.2%)	46 (74.2%)		
	IV	5	0	0	3 (60.0%)	2 (40.0%)		
Differentiation grade								
	I	8	2	5	1 (12.5%)	0 (0.0%)	**0.675**	**0.000**
	II	38	1	7	17 (44.7%)	13 (34.2%)		
	III	57	0	0	5 (8.8%)	52 (91.2%)		
Chemotherapy sensitivity								
	Sensitive	84	3	9	18	54	-0.041	0.666
	Resistant	19	0	3	5	11		

Underlined values show statistical data with significant difference.

Kendall’s tau-b correlation coefficients (r) were applied by the Bivariate Correlation analysis. A P value less than 0.05 was considered significant.

### The expression of ENPP1 protein in epithelial ovarian cancer

**Fig 2:**
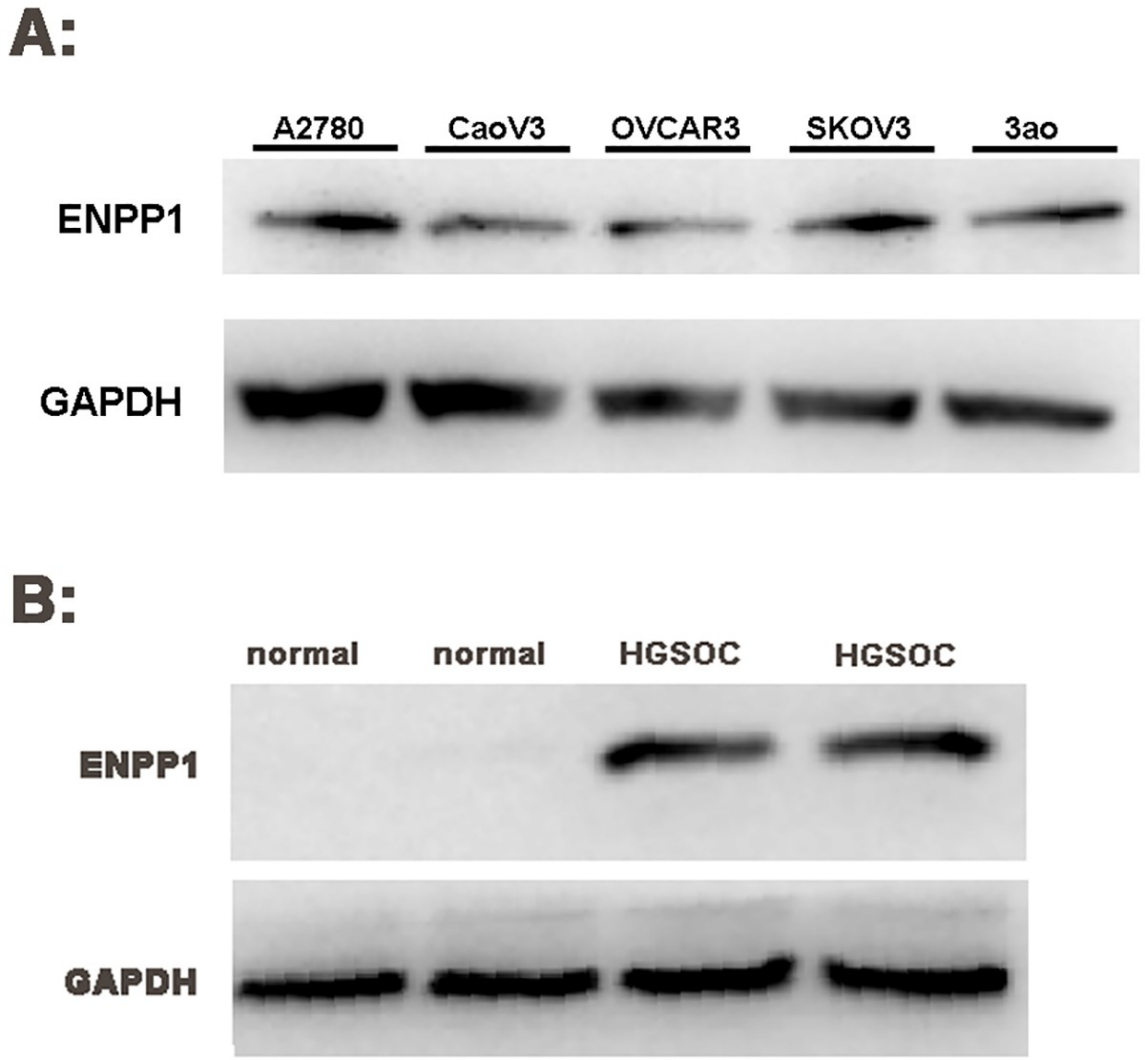
High expression of ENPP1 protein in ovarian epithelial cancer (Western Blot). *A*: ENPP1 protein expression in ovarian epithelial cancer cell lines; *B*: ENPP1 protein expression in ovarian epithelial tissue. A2780: Ovarian epithelial cancer cell line A2780; CaoV3: Ovarian epithelial cancer cell line CaoV3; OVCAR3: Ovarian epithelial cancer cell line OVCAR3; SKOV3: Ovarian epithelial cancer cell line SKOV3; 3ao: Ovarian epithelial cancer cell line 3ao normal: normal ovarian epithelial tissue; HGSOC: high-grade serous ovarian carcinoma epithelial tissue.

Results also showed that ENPP1 gene was highly expressed in A2780, CaoV3, OVCAR3, SKOV3 and 3ao (**Fig 2A)**. The A2780 cell line with strong expression of ENPP1 was used as the target cell for our subsequent RNA interference experiment. As show in **Fig 2B**, the expression of ENPP1 protein in HGSOC was significantly higher than that in normal ovarian tissue (t = 21.562, P = 0.000), suggest that the expression of ENPP1 may be involved in the initiation and development of epithelial ovarian cancer.

### The ENPP1 mRNA and protein expression knockdowned by PC-1 siRNA

After 48h of PC-1 siRNA transfection, A2780 cells were collected to extract RNA and protein for qRT-PCR and Western Blot analysis. We found that the ENPP1 mRNA expression was down regulated by 85.47% after transfection with PC-1 siRNA (Control siRNA compared with Blank, t = 0.627, P = 0.823; PC-1 siRNA compared with Blank, t = 27.487, P = 0.000; PC-1 siRNA compared with Control siRNA, t = 28.489, P = 0.000).

**Fig 3:**
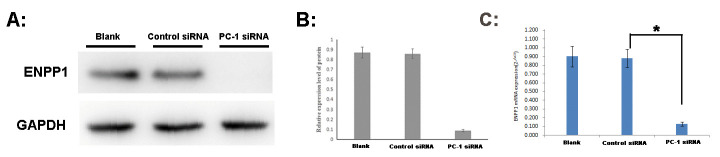
ENPP1 mRNA and protein expression of A2780 after 48h of PC-1 siRNA transfection. *A*: Western Blot electrophoresis; *B*: Analysis of protein relative expression. *C*: qRT-PCR. Blank: A2780 cells was interfered by cell culture medium without any siRNA; Control siRNA: A2780 cells was interfered by Control siRNA; PC-1 siRNA: A2780 cells was interfered by PC-1 siRNA. *P<0.05.

It was also found that the expression of ENPP1 protein decreased significantly, with a decline rate of more than 80% (Control siRNA compared with Blank, t = 0.517, P = 0.619; PC-1 siRNA compared with Blank, t = 31.067, P = 0.000; PC-1 siRNA compared with Control siRNA, t = 32.620, P = 0.000). The results of five repetitions showed statistically significant difference as shown in **Fig 3A–3C**.

### Cell proliferation, caspase 3 activity, migration and invasion with ENPP1 knockdown

**Fig 4:**
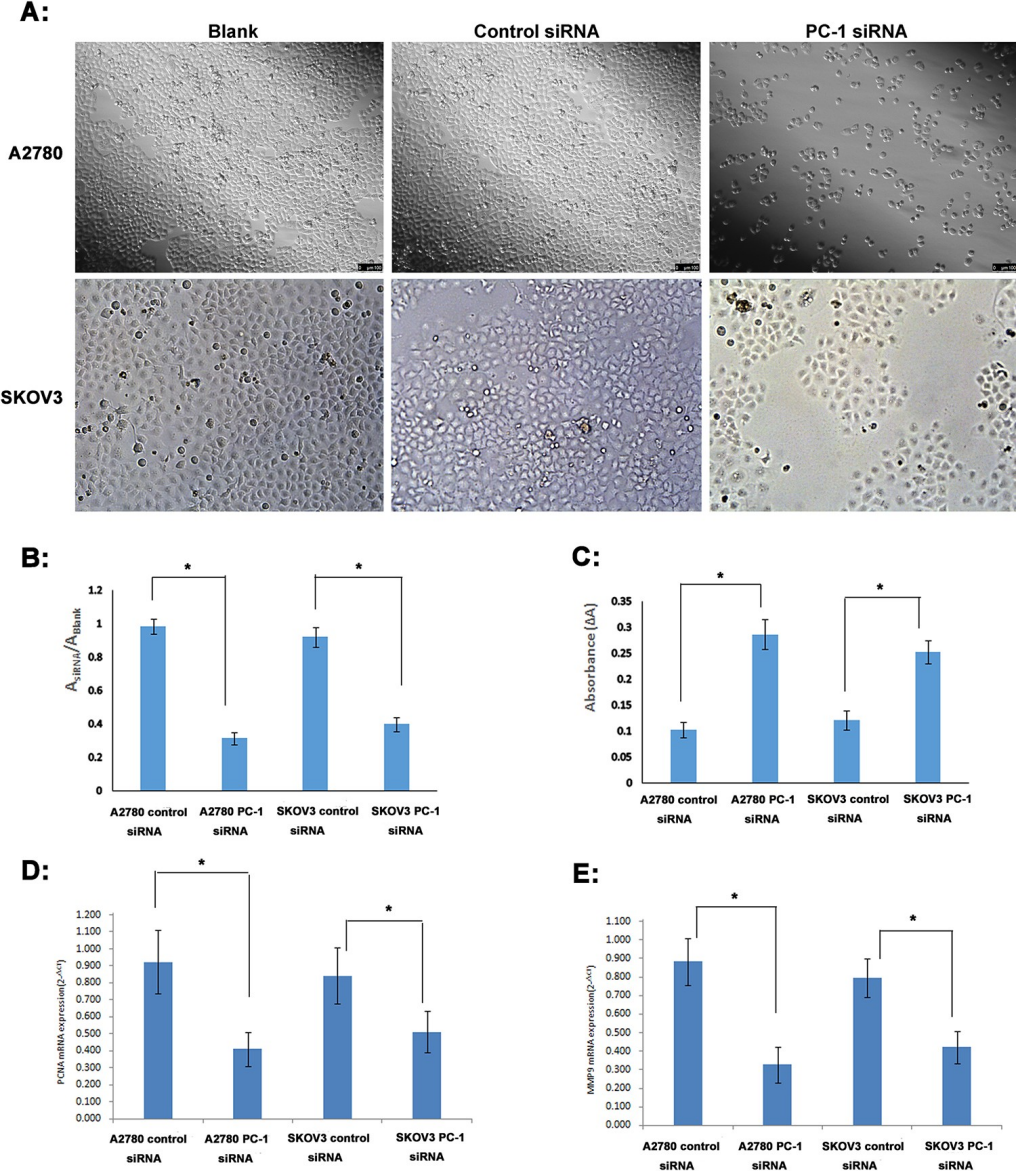
Assays of cell proliferation, caspase 3 activity, PCNA and MMP9 expression after 48h transfection with PC-1 siRNA. *A*: Under the microscope; *B*: BrdU Cell Proliferation assay; *C*: Caspase 3 activity assay; *D*: PCNA mRNA expression (qRT-PCR); *E*: MMP9 mRNA expression (qRT-PCR); A&B: Compared with the control siRNA group, the number of A2780 and SKOV3 cells transfected with PC-1 siRNA was significantly reduced, and the cell proliferation rate decreased significantly; C: Caspase 3 activity of A2780 and SKOV3 cells transfected with PC-1 siRNA was increased 2.79 and 2.07 fold; D: the expression of PCNA mRNA in A2780 and SKOV3 was down regulated by 55.47% and 39.19%; E: the expression of MMP9 mRNA in A2780 and SKOV3 was down regulated by 62.97% and 46.98%. *P<0.05.

Proliferation assay showed that after 48h transfection with PC-1 siRNA, the number of A2780 and SKOV3 cells was significantly reduced about 68.82% and 56.55%. Meanwhile, suspended cells and cell debris of those tumor cells were increased under the microscope (**Fig 4A and 4B**).

We next measured apoptosis of A2780 and SKOV3 cells after 48h transfection with PC-1 siRNA (**Fig 4C**). Caspase 3 activity was increased 2.79 and 2.07 fold in A2780 and SKOV3 cells when PC-1 siRNA group was compared with control siRNA group.

Furthermore, we also detected the expression of PCNA and MMP9 in ovarian cancer cell lines after transfection with PC-1 siRNA. We found that after transfection of PC-1 siRNA, the expression of PCNA mRNA in A2780 and SKOV3 was down regulated by 55.47% and 39.19%, respectively(**Fig 4D**), and the expression of MMP9 mRNA was down regulated by 62.97% and 46.98%, respectively (**Fig 4E**). It further indicated that the proliferation, metastasis and invasion ability of ovarian cancer cells were significantly decreased after down regulating the expression of ENPP1 by PC-1 siRNA.

**Fig 5:**
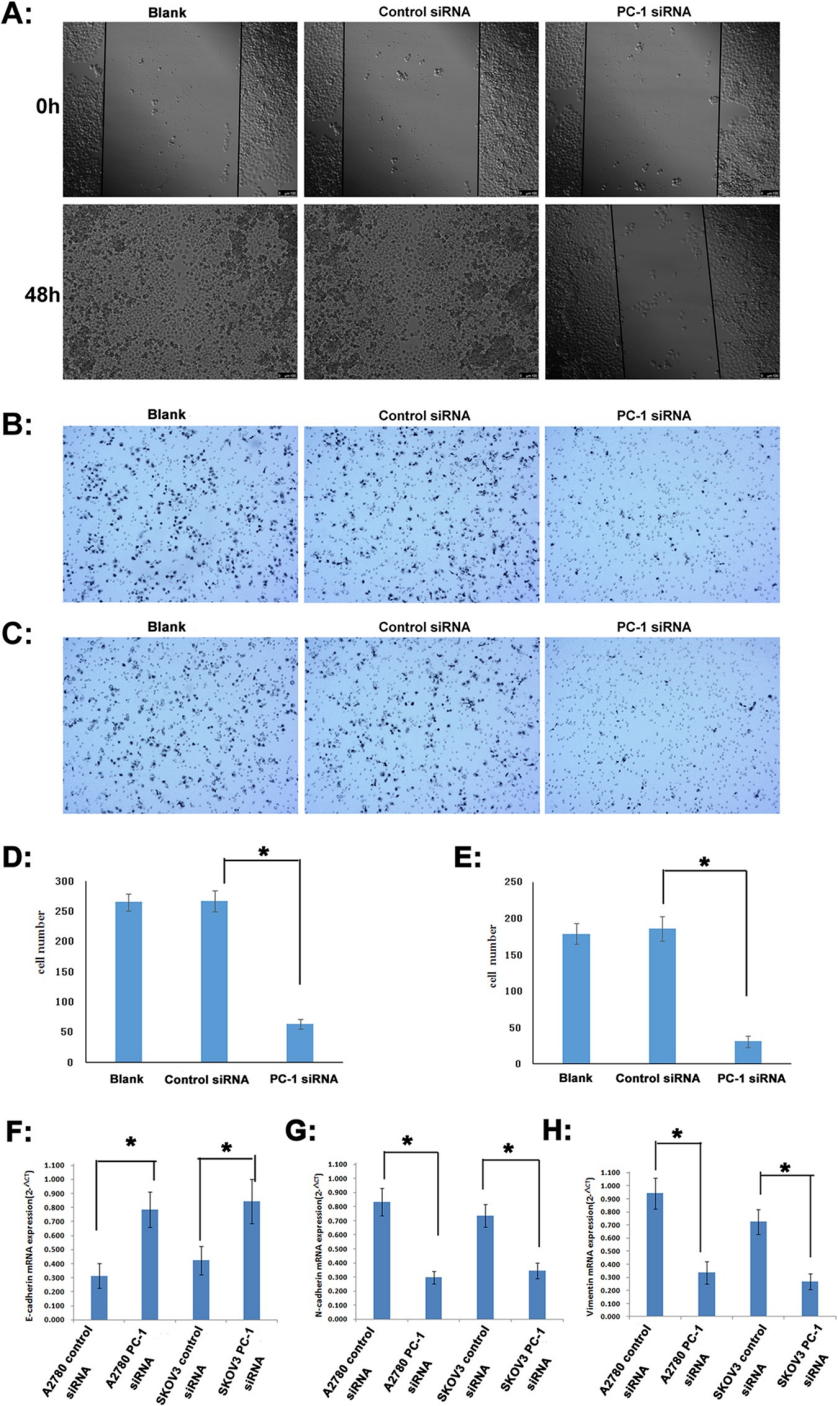
Detection of migration and invasion of A2780 cells after 48h transfection with PC-1 siRNA. *A*: wound-healing assay; *B*: Transwell migration assay; *C*: Matrigel invasion assay; *D*: quantification for transwell migration assay; *E*: quantification for matrigel invasion assay; *F*: E-cadherin mRNA expression (qRT-PCR); *G*: N-cadherin mRNA expression (qRT-PCR); *H*: vimentin mRNA expression (qRT-PCR); *P<0.05.

In addition, change of migratory ability with ENPP1 knockdown was measured with a wound-healing assay. After 48h of scratching, the cells in blank control siRNA group fused together, while the scratch area in PC-1 siRNA group was not reduced significantly. A2780 cells transfected with PC-1 siRNA migrated and filled the wound more slowly than cells transfected control siRNA or with blank (**Fig 5A**).

Consistent with the wound healing, transwell migration/Matrigel invasion assay also showed that ENPP1 knockdown weakened the invasion and migration capacities of A2780 cells. In transwell migration assay, the cell number of the PC-1 siRNA group able to penetrate the chamber was decreased (64±8) compared to those of the control siRNA group (267±17) or blank group(265±14) (**Fig 5B and 5D**). Similarly, in Matrigel invasion assay, the cell number of the PC-1 siRNA group able to penetrate the chamber was decreased (31±4) compared to those of the control siRNA group (186±17) or blank group (179±15) (**Fig 5C and 5E**).

Meanwhile, after ENPP1 was inhibited by PC-1 siRNA, the expression of E-cadherin in A2780 and SKOV3 cells increased by 1.50 and 0.99 times **(Fig 5F)**, and the expression of N-cadherin decreased by 64.35% and 53.26%, respectively **(Fig 5G)**. At the same time, the expression of vimentin also significantly decreased by 64.23% and 62.85%, respectively (**Fig 5H)**, indicating that interference of ENPP1 expression with PC-1 siRNA can effectively inhibit EMT.

## Discussion

ENPP1 is located at 6q22-q23 and is a type II transmembrane glycoprotein with pyrophosphatase and phosphodiesterase activity, expressed highly in bone and cartilage [17, 18]. Biologically, Enpp1 is well known for its role in regulating bone mineralization, serving as the principal ectoenzyme responsible for the generation of extracellular inorganic pyrophosphate (PPi) [17]. In addition, ENPP1 has been shown to modulate insulin signaling, recently reported to regulate bone acquisition and energy metabolism through effects on osteoblasts [19], while also functioning in purinergic signaling [20] and ATP-mediated apoptosis [13, 21]. Recent study shows that, ENPP1 upregulates ABCG2 transporter, increase tumor seeding ability and resistant to conventional chemotherapy in breast cancer [14]. Knockdown of ENPP1 in glioblastoma stem-like cells downregulates stem cell-associated genes, induces tumor differentiation, cell death, and sensitization to chemotherapeutic treatment [15].

Consistent with previous studies in lung cancer and breast cancer, our present study found that ENPP1 protein expression was significantly higher in HGSOC than in ovarian serous cystadenoma, but almost no expression was found in normal ovarian epithelium. Laura et al. found that the expression of ENPP1 in breast cancer cell lines (including MDA-MB-231 and MDA-MB-468) and breast cancer tissues was significantly higher than that in normal breast tissue [22]. Similarly, Hu et al. analyzed four lung cancer data from ONCOMINE and found that ENPP1 expression was significantly up-regulated in lung adenocarcinoma compared with normal lung tissues. Further, they detected the ENPP1 expression in several lung cancer cell lines (including H1792, A549 and HCC827) and found that ENPP1 was also highly expressed [23]. ENPP1 gene may play a key role in maintaining the malignancy and proliferative activity of glioblastoma cells, which has been verified in the study of glioblastoma. Bageritz et al found that in GSCs with ENPP1 gene knocked out, stem cell-related genes were down-regulated (e.g. CD133), stem-like cells also differentiated into mature astrocytic cells, spheroid formation disorders, increased death cells, cell arrest in G1/G0 phase, and drug resistance reversed. These results suggest that ENPP1 is essential for the maintenance of GSCs *in vitro*, and ENPP1 keeps GSCs in undifferentiated and proliferative state [15]. Similarly, our results also showed that ENPP1 was highly expressed in epithelial ovarian cancer cell lines (A2780, CaoV3, OVCAR3, SKOV3 and 3ao). These studies suggest that ENPP1 maybe an initial factor of malignant transformation of normal epithelial cells and plays an important role in maintaining the proliferation activity of cancer cells.

The relationship between prognostic parameters and chemosensitivity with ENPP1 protein expression in patients with HGSOC was also stratified. These indicators include preoperative serum CA125, volume of ascites, tumor maximum size, FIGO stage, cell differentiation grade and chemosensitivity. Our results showed that the expression of ENPP1 was highly correlated with clinical FIGO staging and tumor cell differentiation. The stronger the expression of ENPP1 was, the later the FIGO stage, the worse the cell differentiation. This suggests that with the increase of ENPP1 expression, the invasion and metastasis of cancer cells spread faster, the tumor cell differentiation is poorer. The poorer cell differentiation means the stronger cell proliferation activity, the higher malignant degree. These coincide with previous studies: stem cell characteristics of cancer cells and EMT are closely related to the initiation, progression and metastasis of tumors [24–26]. In the study of lung cancer, it was found that when ENPP1 gene was knocked out in lung cancer cell lines, the expression of a large number of stem cell markers decreased, including ABCG2, SOX2, NANOG, and CD44. Meanwhile, the phenotypic characteristics of EMT induced by TGF-beta were reversed [23]. Similarly, in the study of breast cancer, the expression of ENPP1 in cancer cells at bone metastatic tumor cell was stronger than that in primary tumor cells, and the cloning rate of stable high-expression ENPP1 cell lines was higher than that of non-expression ENPP1 mother cell lines *in vitro* study of bone metastasis [22]. These studies explain our experimental results very well, because of the molecular biological function of ENPP1 gene, ovarian cancer cells with high expression of ENPP1 protein are more prone to metastasis and rapidly proliferate to form metastatic foci, which leads to later stage and indicates poorer prognosis.

In present study, we conducted further cytological experiments on the role of ENPP1 in ovarian cancer cells. After down-regulating the expression of ENPP1, the proliferation, migration and invasion of ovarian cancer cells were significantly decreased, and the expression of proliferation related genes PCNA and metastasis related genes MMP9 were significantly decreased, indicating that the malignant behavior of ovarian cancer cells was inhibited. At the same time, the expression of Caspase-3 was significantly increased, which suggested that the inhibition of cell malignant behavior may be through down-regulating ENPP1 to initiate cell apoptosis.

The process, known as epithelial mesenchymal transition (EMT), plays an important role in tumor development, wound healing and stem cell behavior, and plays an important role in tumor pathological progress, metastasis and drug resistance. Our study found that after ENPP1 was inhibited by PC-1 siRNA, the expression of E-cadherin in A2780 and SKOV3 cells was increased, and the expression of N-cadherin and vimentin were both decreased significantly, indicating that the cellular biological process of EMT is involved in the occurrence and development of ovarian cancer cells. The induction of EMT can make cancer cells lose contact with each other and degrade the local basement membrane by increasing the expression of matrix degrading enzymes to support their migration and invasion. However, interference of ENPP1 expression with PC-1 siRNA can effectively inhibit EMT, thus effectively inhibiting the invasion and metastasis of ovarian cancer.

In summary, compared with normal ovarian epithelium and ovarian serous cystadenoma, the expression of ENPP1 in HGSOC was significantly increased. Moreover, the strong expression of ENPP1 is closely related to FIGO staging and differentiation of tumor cells. The inhibition of malignant behavior and the increase of apoptotic gene expression after the downregulation of ENPP1 suggest that ENPP1 may be a potential target of molecular therapy.

## Supporting information

S1 Raw image (PDF)

## Abbreviations

EOCs: epithelial ovarian cancers
HGSOC: high grade serous ovarian carcinoma
ENPP1: Ecto-nucieotdepyrophosphatase/phosphodesterasel
GSCs: glioblastoma stem cell-like cells
EMT: epithelial mesenchymal transition
IHC: immunohistochemistry
PAGE: polyacrylamide gel
qRT-PCR: Quantitative Real Time-Polymerase Chain Reaction
PCNA: Proliferating Cell Nuclear Antigen
MMP9: Matrix metalloproteinase 9
EMT: epithelial–mesenchymal transition
