# Supplementary material for: High expression of ENPP1 in high-grade serous ovarian carcinoma predicts poor prognosis and as a molecular therapy target
Source: PLoS One. 2021 Feb 26;16(2):e0245733. doi: 10.1371/journal.pone.0245733 (PMC7909685; doi:10.1371/journal.pone.0245733)
Supplement: S1 Raw image — (PDF) [file pone.0245733.s001.pdf]

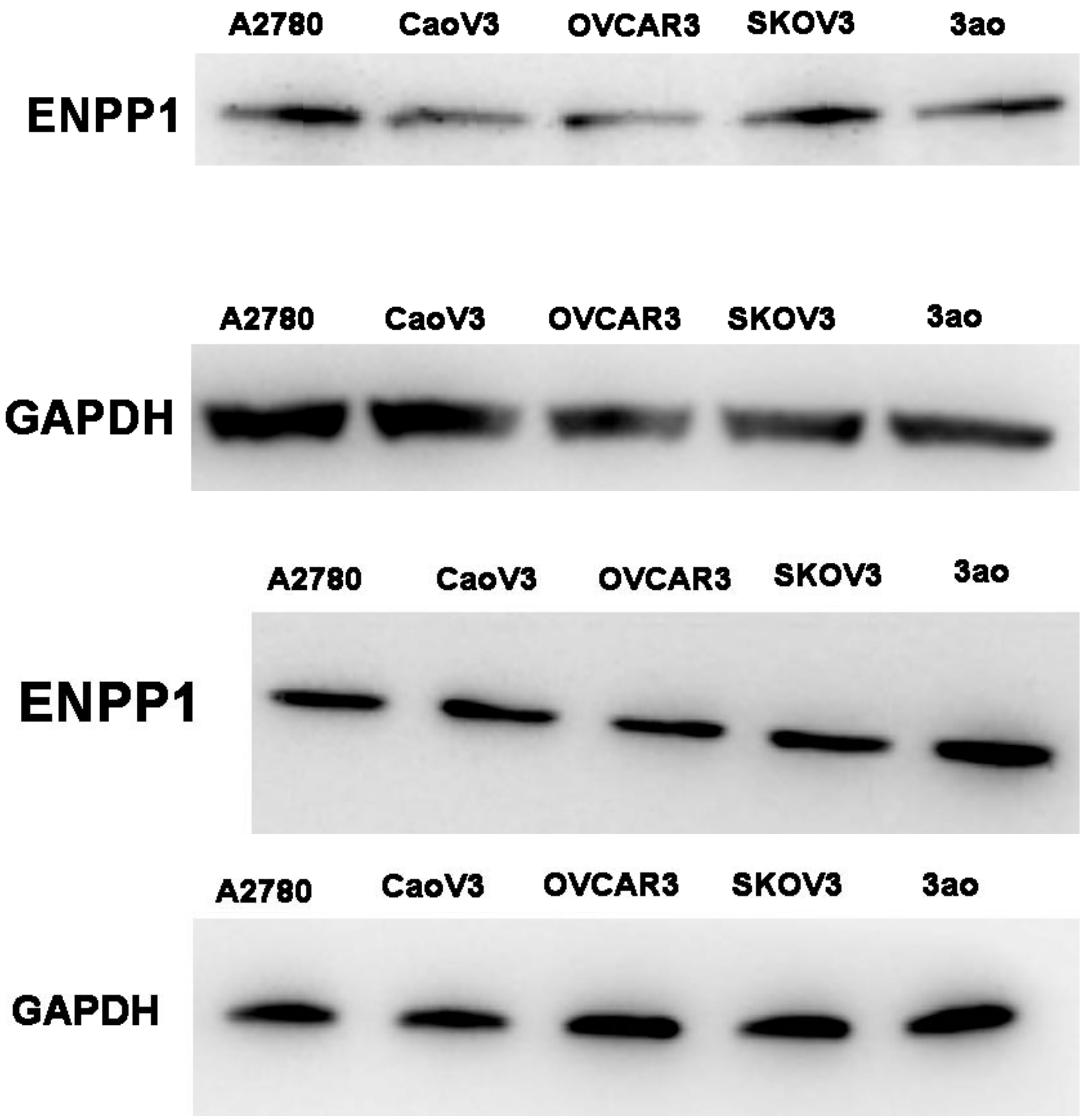
A2780
CaoV3
OVCAR3
SKOV3
3ao
ENPP1
A2780
CaoV3
OVCAR3
SKOV3
3ao
GAPDH
A2780
CaoV3
OVCAR3
SKOV3
3ao
ENPP1
A2780
CaoV3
OVCAR3
SKOV3
3ao
GAPDH

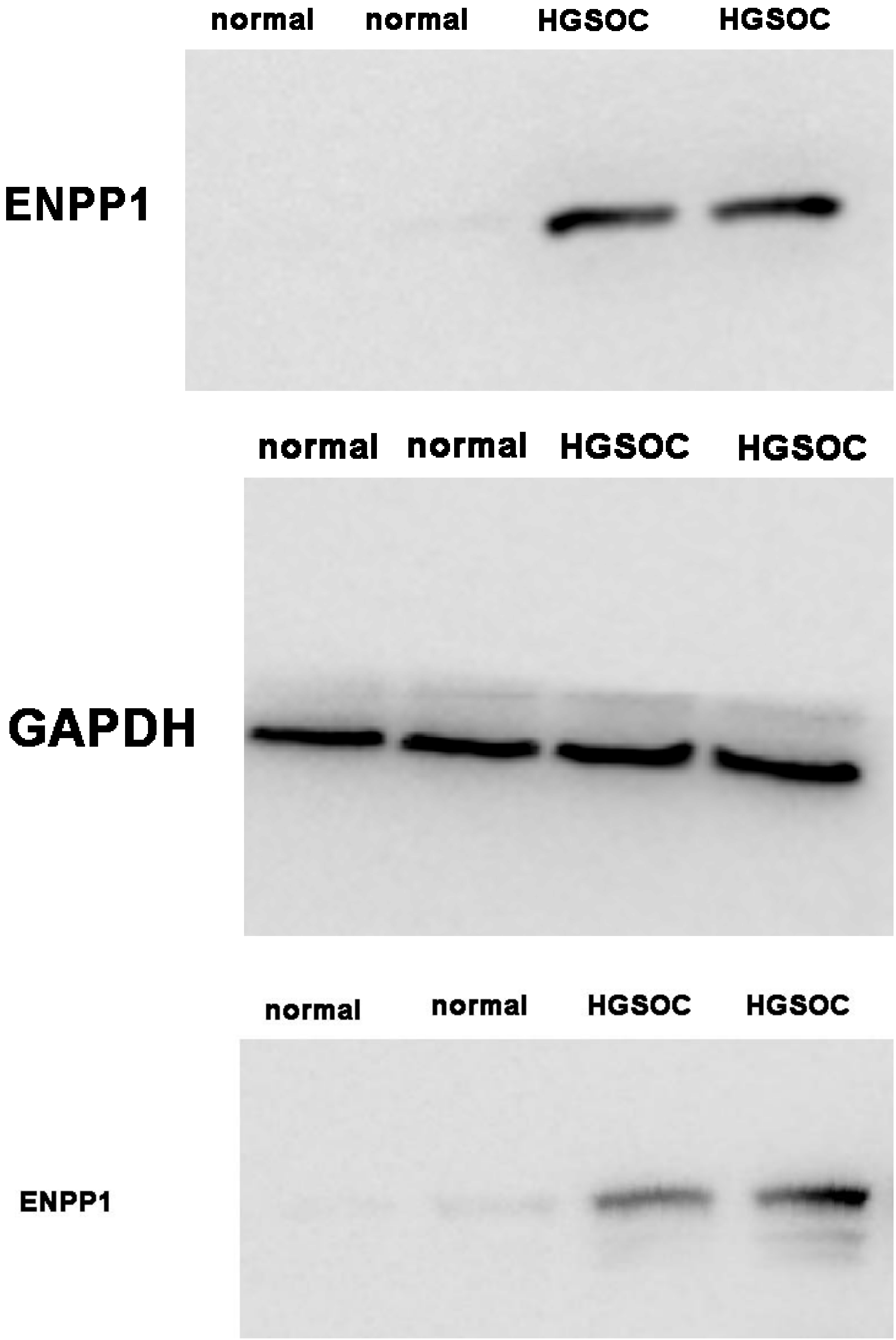
normal
normal
HGSOC
HGSOC
ENPP1
normal
normal
HGSOC
HGSOC
GAPDH
normal
normal
HGSOC
HGSOC
ENPP1

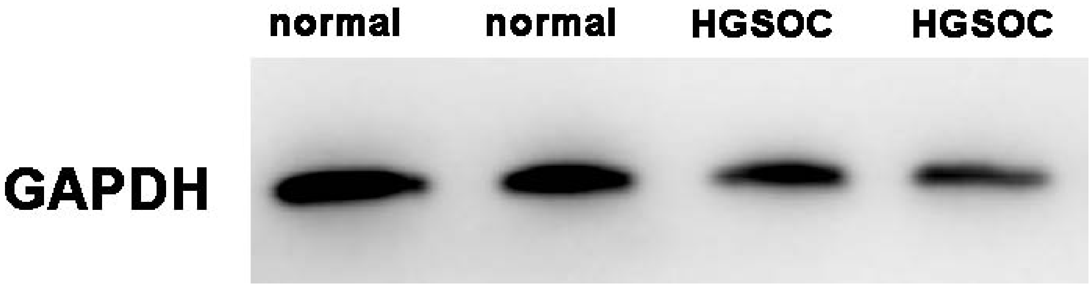

Figure 2. High expression of ENPP1 protein in ovarian epithelial cancer (Western Blot)
A: ENPP1 protein expression in ovarian epithelial cancer cell lines; ***B:*** ENPP1 protein expression in ovarian epithelial tissue

A2780: Ovarian epithelial cancer cell line A2780; **CaoV3:** Ovarian epithelial cancer cell line CaoV3; **OVCAR3:** Ovarian epithelial cancer cell line OVCAR3; **SKOV3:** Ovarian epithelial cancer cell line SKOV3; **3ao:** Ovarian epithelial cancer cell line 3ao
normal: normal ovarian epithelial tissue; **HGSOC:** high-grade serous ovarian carcinoma epithelial tissue

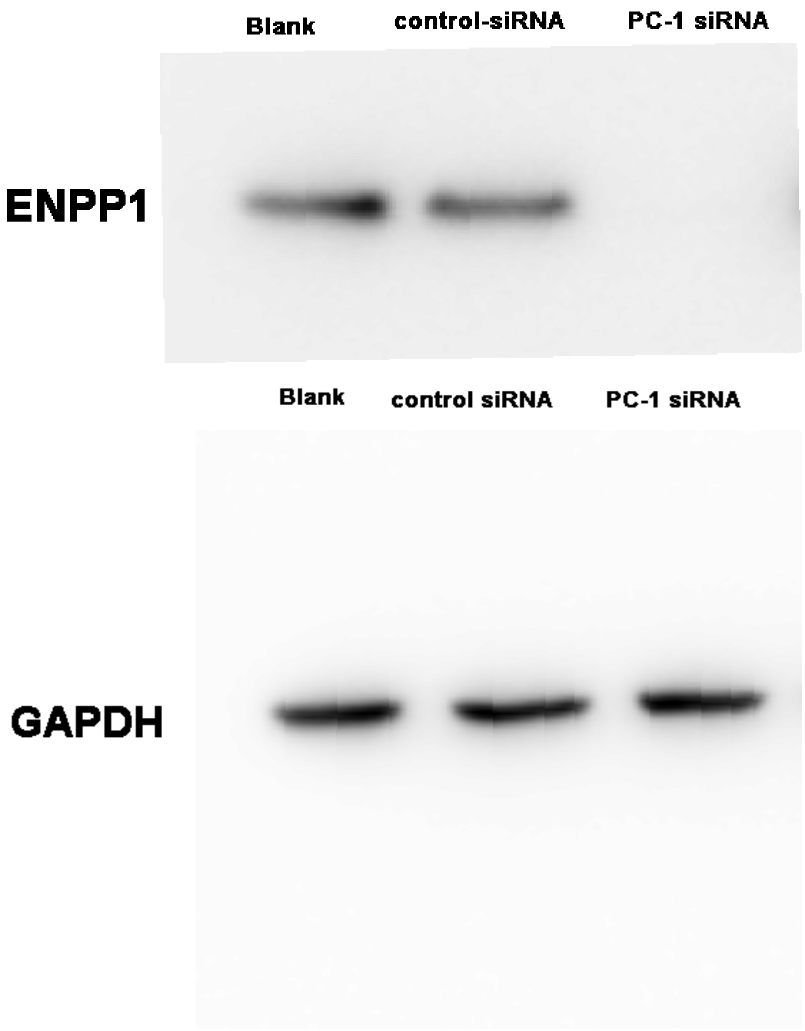
Blank
control-siRNA
PC-1 siRNA
ENPP1
Blank
control siRNA
PC-1 siRNA
GAPDH

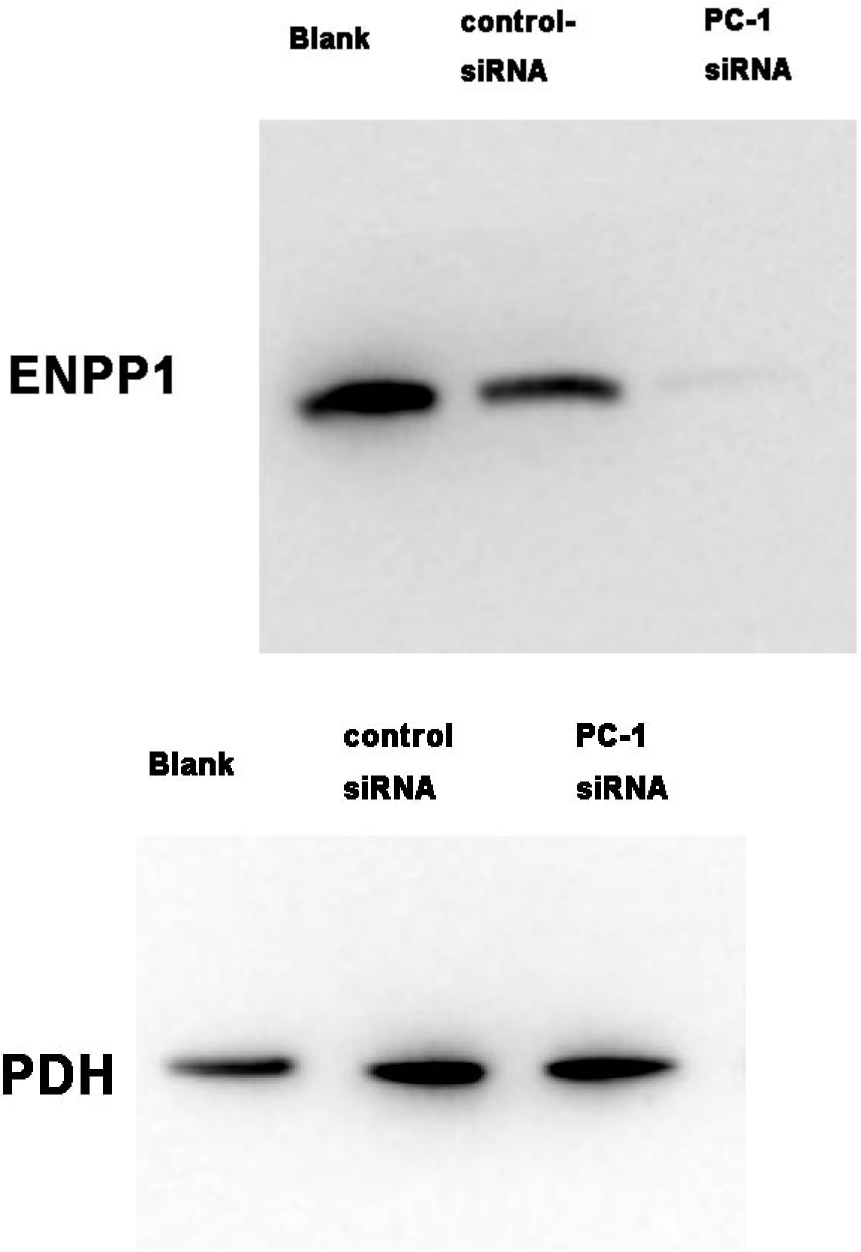

Figure 3. ENPP1 mRNA and protein expression of A2780 after 48h of PC-1 siRNA transfection

A: Westren Blot electrophoresis; ***B:*** Analysis of protein relative expression. ***C:***; qRT-PCR

Blank: A2780 cells was interfered by cell culture medium without any siRNA; **Control siRNA:** A2780 cells was interfered by Control siRNA; **PC-1 siRNA:** A2780 cells was interfered by PC-1 siRNA

*P<0.05
